# A legume product fermented by *Saccharomyces cerevisiae* modulates cutaneous atopic dermatitis-like inflammation in mice

**DOI:** 10.1186/1472-6882-14-194

**Published:** 2014-06-18

**Authors:** Chiou-Yueh Yeh, Chiau-Jing Jung, Ching-Ning Huang, Ying-Che Huang, Huei-Ting Lien, Won-Bo Wang, Li-Fang Wang, Jean-San Chia

**Affiliations:** 1 Graduate Institute of Immunology, College of Medicine, National Taiwan University, No. 1, Jen Ai Road 1st Section, Taipei City 10051, Taiwan; 2 Graduate Institute of Oral Biology, School of Dentistry, National Taiwan University, No.1, Changde St., Taipei City 100, Taiwan; 3 Graduate Institute of Microbiology, College of Medicine, National Taiwan University, No. 1, Jen Ai Road 1st Section, Taipei City 10051, Taiwan; 4 Department of Dermatology, College of Medicine, National Taiwan University, No. 1, Jen Ai Road 1st Section, Taipei 10051, Taiwan

**Keywords:** *Saccharomyces cerevisiae* legume fermented product, Epicutaneous sensitization, Th2 response, Eosinophil, Atopic dermatitis

## Abstract

**Background:**

Isoflavone-containing soy products modulate allergic inflammation in mice. In our previously study, IFN-γ and IL-10 production increased in mice fed with *Saccharomyces cerevisiae* legume fermented product (SCLFP), demonstrating that SCLFP had immunomodulatory activity. In this study, we tested the anti-inflammatory effects of SCLFP in a mouse model of cutaneous atopic dermatitis inflammation induced by epicutaneous sensitization.

**Methods:**

Epicutaneous exposure to protein allergens plus Staphylococcal enterotoxin B induced a T helper (Th)-2–dominant immune response as well as cutaneous atopic dermatitis-like inflammation in BALB/c mice. The thickness of the skin epithelium, eosinophil migration, and T helper responses were determined in patched skin and draining lymph nodes of mice fed with and without SCLFP.

**Results:**

Epicutaneous exposure to protein allergens plus Staphylococcal enterotoxin B induced a T helper (Th)-2–dominant immune response as well as cutaneous atopic dermatitis-like inflammation in BALB/c mice. SCLFP feeding attenuated this cutaneous Th2 response, as evidenced by decreased thickening of the epidermis, less eosinophil infiltration, and lower levels of IL-5, IL-13, and CXCL11 expression compared to controls. Oral administration of SCLFP also modulated Th1 responses in draining lymph nodes, with lower levels of IFN-γ, IL-4, and IL-17 expression.

**Conclusion:**

Oral intake of SCLFP modulated the induced Th2 inflammatory responses in skin and might have potential applications for the prevention and treatment of atopic dermatitis.

## Background

Atopic dermatitis is a common cutaneous inflammatory disease. The etiology of this condition is complex, involving abnormal immunological responses to environmental protein allergens and defective skin barriers [1]. Most atopic dermatitis patients have elevated total serum immunoglobulin (Ig) E levels and specific IgE antibodies to environmental allergens [2]. Acute atopic dermatitis skin lesions exhibit T-helper (Th)-2–dominant inflammation characterized by infiltration of CD4^+^ T cells and eosinophils, as well as increased expression of Th2 cytokines including interleukin (IL)-4, IL-5, and IL-13 [3]. Chronic atopic dermatitis lesions demonstrate a Th2 plus Th1 response and tissue remodeling with increased collagen deposition and dermal thickening [4,5].

In contrast to the highly diversified bacterial population on normal skin, more than 90% of colonized bacteria in atopic dermatitis lesions are *Staphylococcus aureus*[6,7]. *Staphylococcal* enterotoxin B (SEB), a *S. aureus*-derived super-antigen, contributes to the propagation of cutaneous inflammation and has been employed in epicutaneous sensitization to establish murine atopic dermatitis models. Epicutaneous exposure to ovalbumin (OVA)/SEB has been shown to induce elevated antigen-specific serum IgE levels [8] and dermal infiltration of eosinophils, mast cells, and T cells with a mixed Th1/Th2 cytokine and chemokine expression profile. Epicutaneous sensitization with OVA also induces a Th17 and Th9 response [9,10].

Products of soybean fermentation are common food sources in Asian countries. Isoflavones, including genistein and daidzein, are a major component of soy fermented products. These compounds are reported to have anti-inflammatory and antioxidant effects [11]. Miyake *et al.* reported that a high intake of isoflavones may be associated with a reduced prevalence of allergenic rhinitis in humans [12]. Smith *et al.* reported that increased consumption of genistein was associated with better lung function in patients with asthma [13]. Kalhan *et al.* further demonstrated that soy genisteins inhibit eosinophilic airway inflammation [14]. In animal models, many reports have shown that administration of isoflavones has beneficial effects on asthma [15,16]. Genistein has been shown to suppress the development of spontaneous atopic-like dermatitis in NC/Nga mice [17]. Furthermore, topical isoflavones reduce experimental cutaneous inflammation induced by UVB irradiation [11].

Soybean products can be metabolized by intestinal micro-organisms to generate prebiotics. These prebiotics can promote the growth of beneficial bacteria and maintain the integrity of the epithelial barrier of the gut [18]. The fermented legume product *Saccharomyces cerevisiae* legume fermented product (SCLFP), sold as BN222, is prepared by fermentation of soybean milk nutrition broth by *Lactobacillus paracasei* and *Saccharomyces cerevisiae*. This product has been used for human dietary supplementation world-wide for several years. In this study, we ask whether this fermented soy-derived-fermented food can modulate a T helper response to alleviate cutaneous inflammation.

## Methods

### SCLFP preparation

SCLFP (sold as BN222), a fermentation product of a mixture of soybeans, black beans, and green beans, was provided by AKC Nutraceuticals Inc., British Columbia, Canada. The microorganisms used in the fermentation process were *Lactobacillus paracasei* and *Saccharomyces cerevisiae*. The nutrient broth used in fermentation was prepared by mixing organic beans (mainly soybeans, black beans, and green beans) with distilled water, followed by grinding, and heat sterilizing (at 100°C). The microorganisms were inoculated into the mixed bean nutrient broth (1.08 x10^8^ CFU/mL) and incubated for 150 h. The final fermented broth was then heat-sterilized, filtered, concentrated, and spray dried. The resulting pathogen-free powders were then packaged into capsules. SCLFP contains oligosaccharides, including fucose, rhamnose, glucosamine, galactose, glucose, mannose, and xylose. The composition of SCLFP is 83.37% isoflavones, including 70.1% daidzein, 7.16% genistein, 2.15% genistin and 3.29% daidzin (Table 1).

**Table 1:** **Composition of *Saccharomyces cerevisiae *** **legume fermented product**

	**Fraction of composition (%)**		
Nutritional composition			
Carbohydrate	22%	Oligosaccharides containing fucose, rhamnose, glucosamine, galactose, glucose, mannose, and xylose	
Protein	43%		
Fiber	18%		
Fat	7%		
Total	83.37%		
Isoflavone composition^1,2^			
Daidzein	70.1%		
Genistein	7.16%		
Genistin	2.15%		
Daidzin	3.29%		

^1^SCLFP composition was analyzed by HPLC/DAD (SGS, Taiwan Ltd); ^2^data are presented as % of total isoflavone content.

### Murine model splenocyte stimulation and epicutaneous sensitization

Female BALB/c mice (6–8 weeks old) were purchased from the animal center of National Taiwan University, College of Medicine (Taipei City, Taiwan) and kept in a specific pathogen-free environment. All animal experiments were approved by the animal care committee of the Medical College of National Taiwan University. Splenocytes were isolated from mice fed with SCLFP (3, 6, or 15 mg) 5 days a week for 4 weeks. The splenocytes were stimulated with 1 μg Concanavalin (Con)A for 3 days, the culture supernatant were collected for cytokine determination. Mice were separated into 3 experimental groups: 1) sham group, which received epicutaneous sensitization with SEB without OVA and were given water; 2, 3) immunization group and immunization + SCLFP group, respectively, both of which received epicutaneous sensitization with OVA + SEB and were given water (immunization group) or SCLFP (immunization + SCLFP group). Briefly, mice were fed with 200 μL of water or 150 mg/mL SCLFP 3 times per week for 8 successive weeks. For epicutaneous sensitization, 20 μL of 100 mg/mL OVA (Sigma-Aldrich, St. Louis, MO, USA) and 10 μL of 400 μg/mL SEB (Sigma-Aldrich, St. Louis, MO, USA) were placed on the disc of a Finn chamber (Epitest, Tuusula, Finland). The discs were then applied to an area of shaved skin on the back of each mouse. For each course of epicutaneous sensitization, freshly prepared patches were applied daily for 5 successive days. Epicutaneous immunizations were performed at weeks 2, 5, and 8 after the beginning of feeding.

### Histological analysis

Skin specimens were obtained from patched areas 8 days after the third sensitization course and fixed in 10% buffered formalin and embedded in paraffin. Multiple 5-μm sections were stained with hematoxylin and eosin (H & E) for evaluation of epidermal thickness and eosinophil counting. The number of eosinophils was counted in 15 high-power fields at 200 × magnification (Zeiss, Axio Imager A1) and presented as cells per high-power field, with mean and standard error of the mean (SEM) calculated.

### RNA preparation

The skin or draining lymph node (DLN) samples were obtained 8 days after the third sensitization course. Cell suspensions of pooled (5 mice per group) DLN cells were prepared from sensitized mice in RPMI 1640 medium (Thermo Scientific Nunc) supplemented with 10% fetal bovine serum and antibiotics. Cells were cultured in the presence of 50 μg/mL OVA and harvested after 24 h. After homogenization, the total RNA was extracted and complementary DNA was synthesized from 2 μg of DNase-I–treated total RNA. Real-time PCR was performed using a KAPA SYBR FAST qPCR kit (Kappa Biosystems, Boston, MA, USA) in a 7500 Real-Time PCR system (Applied Biosystems, Carlsbad, CA, USA). The relative cytokine mRNA expression level of each sample was normalized according to its β-actin expression. The primer used in this study is listed in Additional file 1: Table S1.

### ELISA

Sera were collected 8 days after the third epicutaneous sensitization and stored at −20°C until further analysis. OVA-specific antibody levels were determined by ELISA. Briefly, ELISA plates were coated with 0.5 μg of OVA per well. After washing and blocking, the plates were incubated with serial dilutions of serum samples for 18 h at 4°C. Next, the plates were washed and incubated with biotin-conjugated antibodies directed against mouse IgG1, IgG2a, or IgE (Bethyl Laboratories, Montgomery, TX, USA) for 18 h at 4°C. After washing, samples were treated with streptavidin-conjugated horseradish peroxidase followed by tetramethylbenzidine substrate (Clinical Science Product Inc., Mansfield, MA, USA) for detection. Interferon-γ, IL-10, IL-2, and IL-4 in serum were determined using the DuoSet ELISA Development System (R&D, Systems, Inc.).

### Statistical analysis

Data are presented as the mean ± standard error of the mean (SEM) and performed with one-way analysis of variance (ANOVA) followed by Tukey’s multiple comparison test to compare the mean levels of cytokine expression following a particular treatment. All *p*-values less than 0.05 were considered statistically significant.

## Results

### Immunomodulatory effects of SCLFP administration

After 4 weeks of SCLFP administration, the mice were scarified, the spleens harvested, and single-cell suspensions were prepared for ConA stimulation. Interferon-γ and IL-10 protein expression increased dose-dependently with SCLFP administration (Additional file 2: Figure S1A and B). Expression of IL-2 and IL-4, the Th2-response cytokines were on the decrease (Additional file 2: Figure S1C and D). These results demonstrated SCLFP had immune-modulatory potential that drive immune response to Th1 and immunosuppressive response. Therefore, the Th2 epicutaneous sensitization mice model was selected to test this hypothesis.

### Oral SCLFP administration with epicutaneous sensitization attenuated cutaneous inflammation

**Figure 1:**
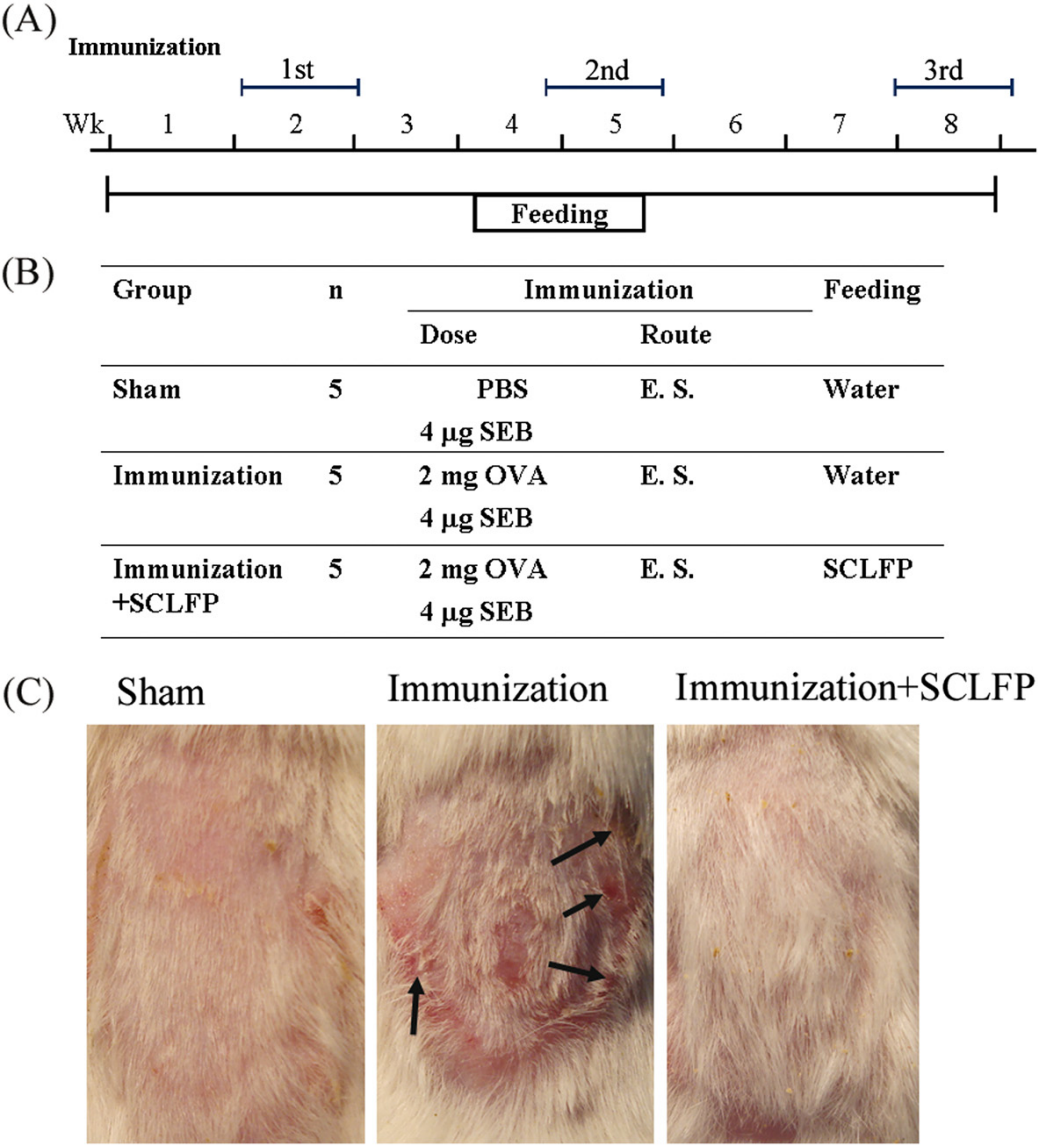
**Epicutaneous sensitization mouse model. (A)** The protocol for epicutaneous sensitization with OVA/SEB and administration of SCLFP. **(B)** Mice received either epicutaneous sensitization with PBS plus SEB and water (sham), sensitization with OVA + SEB and water (immunization), or with SCLFP (immunization + SCLFP). **(C)** The appearance of patched skin after 3 courses of epicutaneous sensitization. Arrows indicate skin lesions.

**Figure 2:**
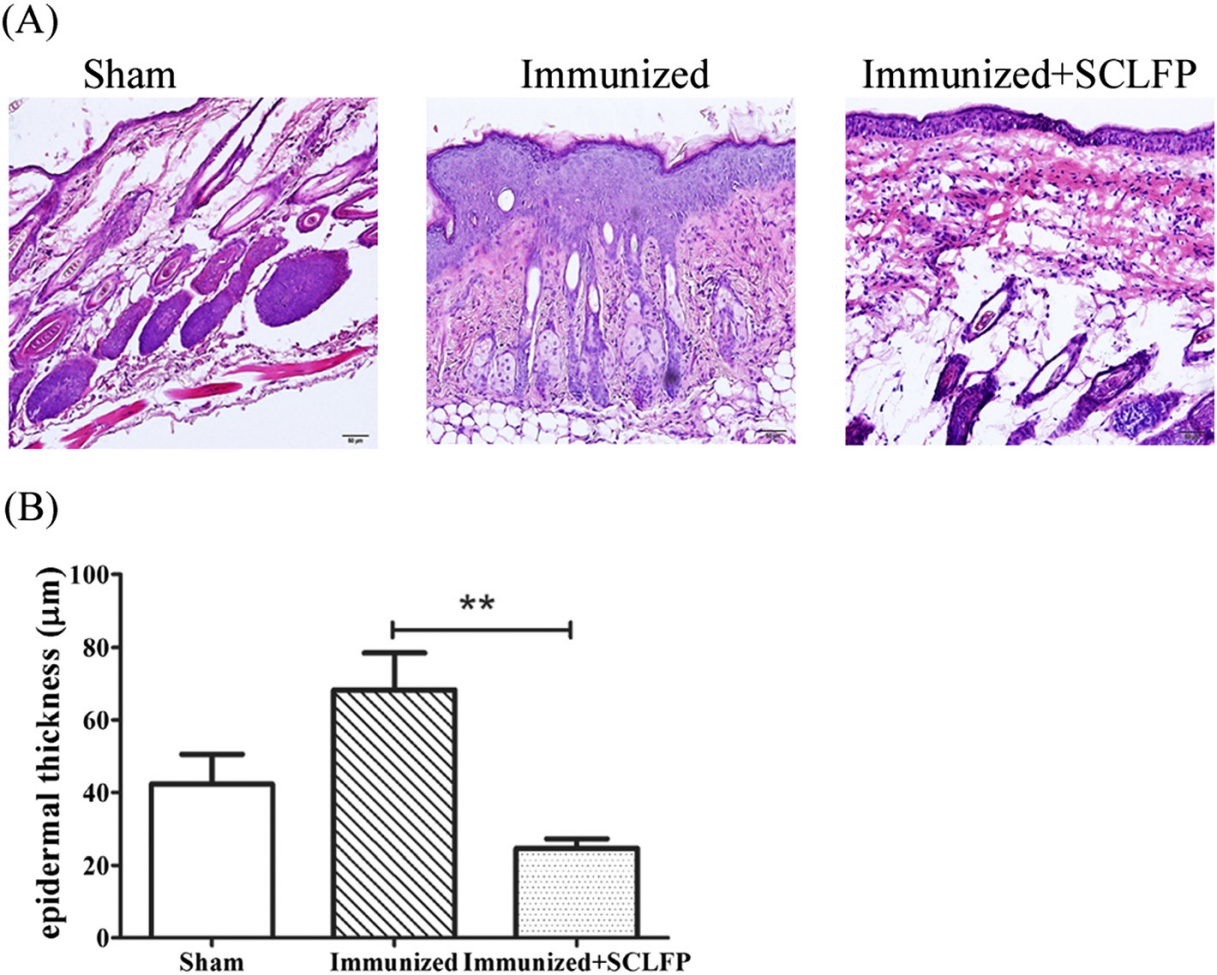
**Histological features of patched skin in BALB/c mice.** Mice were epicutaneously sensitized and fed as shown in Figure 1. **(A)** Marked thickening of the epidermis with mild spongiosis and inflammatory cell infiltration in the dermis. **(B)** Epidermal thickness was determined in each of the 3 groups of mice. Data are presented as the mean ± standard error of the mean (SEM) and performed with one-way analysis of variance (ANOVA) followed by Tukey’s multiple comparison test to compare each group. All *p*-values less than 0.05 were considered statistically significant.

The effects of oral SCLFP administration on cutaneous allergic inflammation were explored using the murine epicutaneous sensitization model (Figure 1A,B). As expected, mice receiving an epicutaneous OVA/SEB patch (immunized group) developed remarkable erythema with scaling and crust formation on the patched skin (Figure 1C). In contrast, mice fed SCLFP daily showed significant attenuation of cutaneous inflammation (Figure 1C). Mice receiving epicutaneous sensitization with phosphate-buffered saline (sham group) showed only marginal erythema (Figure 1C). Histological features were also examined (Figure 2A). In the immunized group, the patched skin showed acanthosis, spongiosis, and inflammatory cell infiltration of the dermis. The epidermis was markedly thickened (68.21 ± 10.20 μm) in the immunized group compared to that of the sham group (42.29 ± 8.180 μm). SCLFP administration significantly reduced the thickening of epidermis (24.55 ± 2.644 μm; *p* = 0.0061) (Figure 2B). These results demonstrated that treatment with SCLFP attenuated the cutaneous inflammation induced by epicutaneous OVA/SEB sensitization.

### SCLFP administration with epicutaneous sensitization attenuated eosinophil infiltration and Th2 cytokine expression

**Figure 3:**
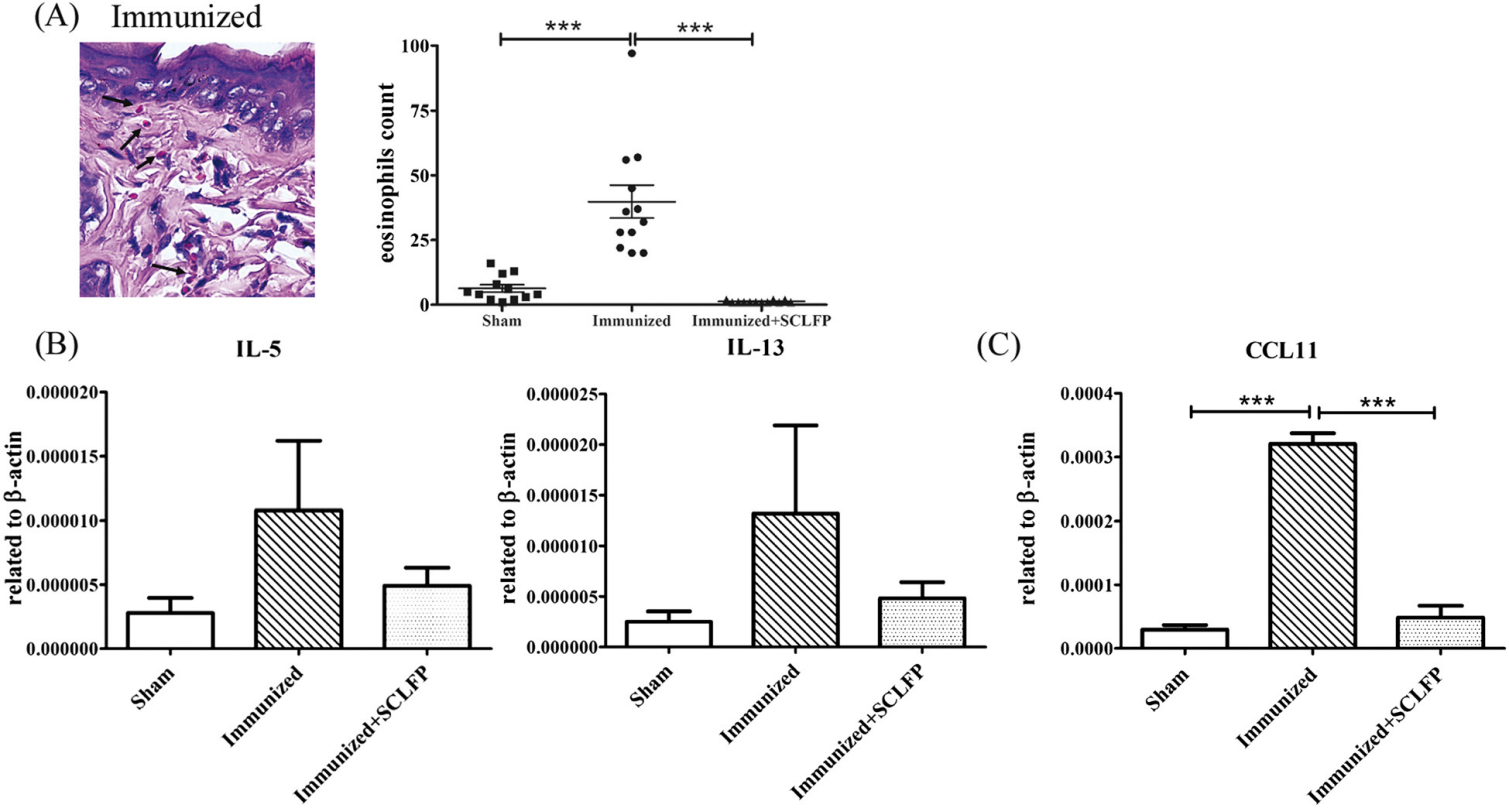
**The Th2-induced cutaneous inflammation was attenuated with oral administration of SCLFP.** Mice were treated as shown in Figures 1 and 2. **(A)** Eosinophil counts in induced cutaneous inflammation; H&E staining. **(B, C)** Expression levels of IL-5 and IL-13 **(B)** and CCL11 **(C)** in induced cutaneous inflammation. Data are presented as values normalized to β-actin expression and presented as the mean ± SEM and performed with one-way analysis of variance (ANOVA) followed by Tukey’s multiple comparison test to compare each group. All *p*-values less than 0.05 were considered statistically significant.

The number of eosinophils in the immunized group increased markedly (39.83 ± 6.348 per high-resolution field, HRF) compared to that of the sham group (6.333 ± 1.411 per HRF; *p* = 0.0002) (Figure 3A). Notably, the number of eosinophils in the SCLFP group was much lower (1.250 ± 0.1306 per HRF) than that of the immunized group (*p* < 0.0001).

The Th2 expression levels of IL-5 and IL-13 in induced cutaneous inflammation of the immunized group markedly increased over that of controls. The expression levels of IL-5 and IL-13 were significantly lower in the SCLFP group than in the immunized group (IL-5, *p* = 0.0132; IL-13, *p* = 0.0130) (Figure 3B). The expression level of CCL11, a Th2 chemokine for eosinophil migration, was much lower in the SCLFP group than in the immunized group (Figure 3C). Furthermore, Th2-related antigen-specific antibody production, including IgG1, and IgE, increased in the immunized group (Additional file 3: Figure S2). SCLFP administration had no effect on the production of these antibodies. Collectively, epicutaneous sensitization with OVA/SEB induced a Th2-predominant cutaneous inflammation that could be attenuated by oral co-administration of SCLFP, but did not affect IgE production.

### SCLFP administration modulates T helper responses in draining lymph nodes

**Figure 4:**
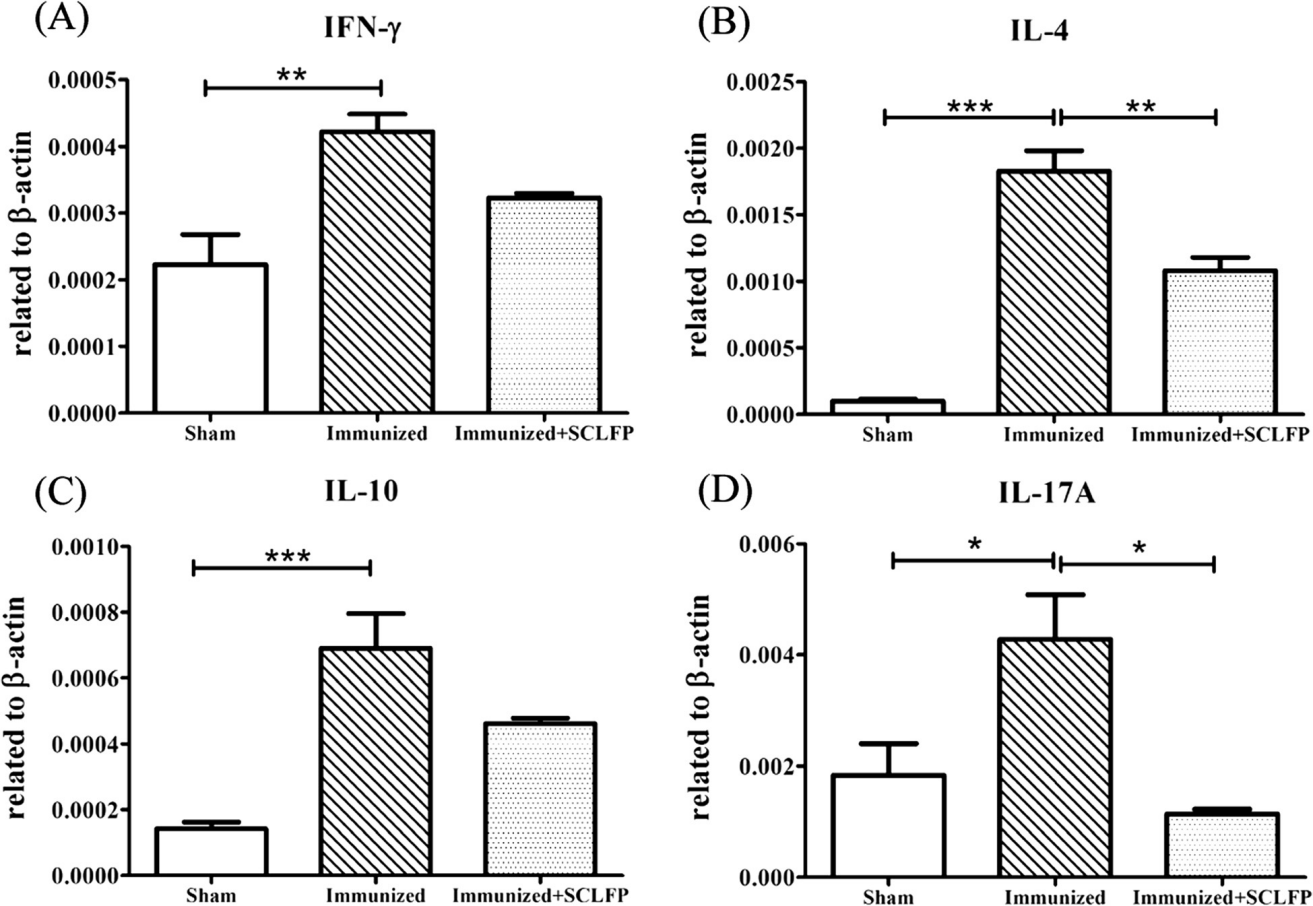
**Cytokine expression in draining lymph nodes.** Mice (n = 5) were treated as shown in Figure 3. DLN were obtained and pooled one week after the third sensitization course. DLN cells were cultured in the presence of 50 μg/mL OVA. Cells were harvested after 24 h for RNA and cDNA preparation. Expression levels of **(A)** IFN-γ, **(B)** IL-4, **(C)** IL-10, and **(D)** IL-17A were measured using quantitative PCR. Data are represented the mean ± SEM and performed one-way analysis of variance (ANOVA) followed by Tukey’s multiple comparison test. All p-values less than 0.05 were considered statistically significant.

Epicutaneous sensitization with a protein antigen induced predominant Th2, a modest Th17, and a marginal Th1 response [8,10,19]. The expression level of IL-4 and IL-10 in draining lymph nodes (DLN) was highly elevated in the immunized group compared to that of the sham group (Figure 4), while interferon (IFN)-γ and IL-17A expression levels were only modestly elevated. Administration of SCLFP to immunized mice significantly decreased the expression of IL-4, and IL-17A in DLN (*p* = 0.007, *p* = 0.012, respectively). The expression of IL-10 and interferon –γ also showed a tendency to decrease, but the difference did not reach statistical significance. These results supported that administration of SCFLP could modulate the Th immune responses induced by epicutaneous sensitization.

## Discussion

In this study, we demonstrated that SCLFP treatment attenuated cutaneous inflammation, with decreased eosinophil infiltration and Th2 cytokine expression, is in agreement with a report by Sakai *et al.,* who observed that genistein administration decreased skin inflammation in atopic-like dermatitis [17]. However, the administration of isoflavones decreased Th2 cytokine expression in DLN in our study; this result contrasts that of the Sakai study, which reported no difference in serum IgE levels between genistein-treated and control mice. The reason for this discrepancy is not clear at present, but we speculate 2 possibilities. First, the isoflavones we administered (SCLFP) differ from the genistein used in the Sakai study. Second, their study used a specific mouse strain that develops atopic dermatitis-like cutaneous inflammation spontaneously, implying an intrinsic abnormal immune response that might not be reversed by isoflavones. Notably, our results are consistent with most reports on this topic, which demonstrate that isoflavones suppress the induced Th2 response in asthma and food hypersensitivity models [20].

Epicutaneous sensitization with protein antigen induces a predominant Th2 response that is crucial for the development of atopic dermatitis. The development of methods or strategies to suppress induction of this Th2 response is very important clinically. Using the murine protein-patch model for epicutaneous sensitization, we have observed that most manipulations, including co-administration of Toll-like receptor (TLR) agonists, irritants, or haptens enhanced the Th2 response (unpublished results). Of all the agents we have tested, only low-energy visible light and co-administration of dectin-1 agonist suppressed the Th2 response [21,22]. Saegusa *et al.* reported that galectin-3 deficient mice exhibited a markedly lower Th2 response to epicutaneous sensitization with protein antigen compared with wild type mice [23]. Kypriotou *et al.* recently demonstrated that activin A, a member of the TGF-β superfamily, inhibited antigen-induced cutaneous Th2 polarization [24]. Thus, our observation that SCLFP suppressed the Th2 response might have potential clinical applications for preventing and treating atopic dermatitis.

The mechanisms underlying the modulatory effects of isoflavones are under investigation. Genistein is structurally similar to estradiol and has been suggested to act as an E2 agonist or antagonist [20] and is also known to be a protein tyrosine kinase inhibitor [16]. Masilamani *et al.* showed that soybean isoflavones regulate dendritic cell function by inhibiting their maturation by altering co-stimulator expression and cytokine secretion [25]. Zhang *et al.* demonstrated that soy isoflavones down-regulate the Th2 response and promote the Th1 response [26]. Kalhan *et al.* demonstrated that genistein also inhibits eosinophil leukotriene C4 synthesis [14].

Soybean products can be metabolized to generate prebiotics that might modulate immune responses through changing the propagation of beneficial bacteria [27]. Our preliminary experiments showed that oral administration of SCLFP increased the amounts of *Lactobacilli* in feces after epicutaneous sensitization (data not shown). Therefore, SCLFP also has the potential to modulate beneficial flora populations in the gut, although the mechanism and extent of such effects needs further confirmation.

## Conclusions

In summary, this study provides evidence that oral administration of SCLFP modulates cutaneous atopic dermatitis-like inflammation in mice and might have potential applications for the prevention and treatment of atopic dermatitis.

## Abbreviations

SCLFP: *Saccharomyces cerevisiae* legume fermented product; Th: T helper; AD: Atopic dermatitis; SEB: Staphylococcal Enterotoxin B; DLN: draining lymph node; OVA: Ovalbumin.

## Competing interests

All authors declare no conflicts of interest and agree to submit this study for publication.

## Authors’ contributions

The contributions of all authors are as follows: CYY and LFW contributed to experimental design, practice, and manuscript preparation; CJJ and WBW assisted in data analysis and discussing results; CNH, YCH, and HTL performed animal experiments; JSC was the supervisor and coordinator. All authors read and approved the final manuscript.

## Pre-publication history

The pre-publication history for this paper can be accessed here:

http://www.biomedcentral.com/1472-6882/14/194/prepub

## Supplementary Material

Additional file 1: Table S1 Primer list. Click here for file

Additional file 2: Figure S1 Immunomodulatory effect of SCLFP administration in splenocytes. Mice were administered the indicated dose of SCLFP once daily, 5 days per week. After 4 weeks, the mice were sacrificed, and single-cell suspensions of spleen were prepared for Concanavalin A stimulation for 3 days. The cultured supernatant was collected for IFN-γ (A), IL-10 (B), IL-2 (C), and IL-4 (D) determination. Data are represented the mean ± SEM and performed one-way analysis of variance (ANOVA) followed by Tukey’s multiple comparison test. All p-values less than 0.05 were considered statistically significant. Click here for file

Additional file 3: Figure S2 Serum antigen-specific antibody responses to administration of SCLFP. Sera were collected 8 days after the third epicutaneous sensitization, and OVA-specific (A) IgG1, (B) IgG2a and (C) IgE antibody levels were determined by ELISA. Data are represented the mean ± SEM and performed one-way analysis of variance (ANOVA) followed by Tukey’s multiple comparison test. All p-values less than 0.05 were considered statistically significant. Click here for file
