# Supplementary material for: A legume product fermented by Saccharomyces cerevisiae modulates cutaneous atopic dermatitis-like inflammation in mice
Source: BMC Complement Altern Med. 2014 Jun 18;14:194. doi: 10.1186/1472-6882-14-194 (PMC4074418; doi:10.1186/1472-6882-14-194)
Supplement: Additional file 1: Table S1 — Primer list. [file 1472-6882-14-194-S1.docx]

**Table S1 Primer list**

| Mouse β-actin | F | 5′-TGTATGAAGGCTTTGGTCTCCCT-3′ |
| --- | --- | --- |
|  | R | 5′-AGGTGTGCACTTTTATTGGTCTCAA-3′ |
| Mouse IFN-γ | F | 5′-CAAGTGGCATAGATGTGGAAG-3′ |
|  | R | 5′-GAAGAAGGTAGTAATCAGGTG-3′ |
| Mouse IL-4 | F | 5′-TGTCATCCTGCTCTTCTTTCTC-3′ |
|  | R | 5′- TCTGTGGTGTTCTTCGTTGC-3 |
| Mouse IL-10 | F | 5′- GGTTGCCAAGCCTTATCGG-3′ |
|  | R | 5′- TCTTCACCTGCTCCACTGC-3′ |
| Mouse IL-5 | F | 5′- AGCACAGTGGTGAAAGAGACCTT-3′ |
|  | R | 5′-TCCAATGCATAGCTGGTGATTT-3′ |
| Mouse IL-13 | F | 5′-AGACCAGACTCCCCTGTGCA-3′ |
|  | R | 5′-TGGGTCCTGTAGATGGCATTG-3′ |
| Mouse CCL11 | F | 5-CAGATGCACCCTGAAAGCCAT-3 |
|  | R | 5-TGCTTTGTGGCATCCTGGAC-3 |
| Mouse IL-17A | F | 5-GGACTCTCCACCGCAATGA-3 |
|  | R | 5-GGCACTGAGCTTCCCAGATC-3 |
