# Supplementary figures and images for: A legume product fermented by Saccharomyces cerevisiae modulates cutaneous atopic dermatitis-like inflammation in mice
Source: BMC Complement Altern Med. 2014 Jun 18;14:194. doi: 10.1186/1472-6882-14-194 (PMC4074418; doi:10.1186/1472-6882-14-194)

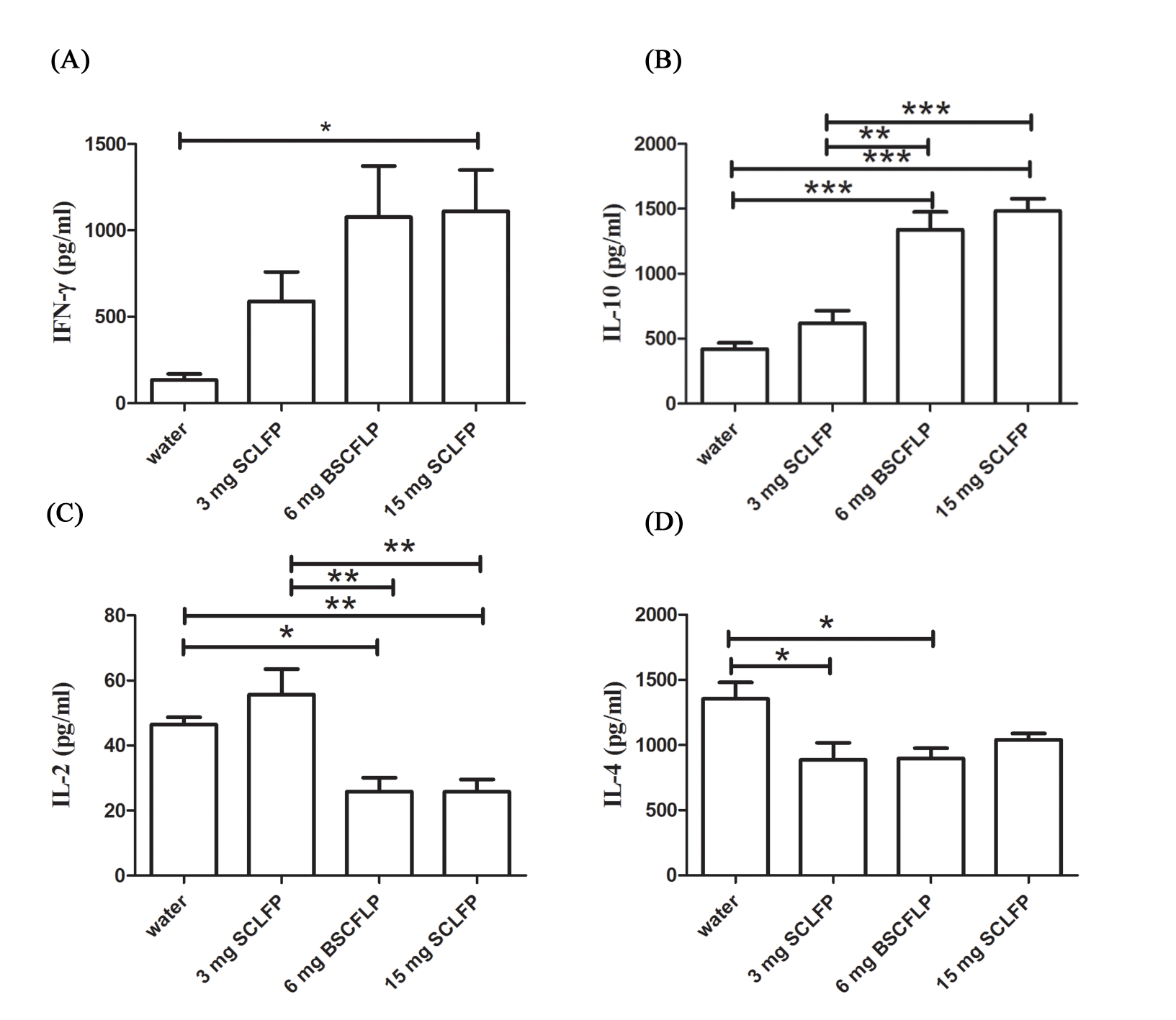

Supplement: Additional file 2: Figure S1 — Immunomodulatory effect of SCLFP administration in splenocytes. Mice were administered the indicated dose of SCLFP once daily, 5 days per week. After 4 weeks, the mice were sacrificed, and single-cell suspensions of spleen were prepared for Concanavalin A stimulation for 3 days. The cultured supernatant was collected for IFN-γ (A), IL-10 (B), IL-2 (C), and IL-4 (D) determination. Data are represented the mean ± SEM and performed one-way analysis of variance (ANOVA) followed by Tukey’s multiple comparison test. All p-values less than 0.05 were considered statistically significant. [file 1472-6882-14-194-S2.tiff]

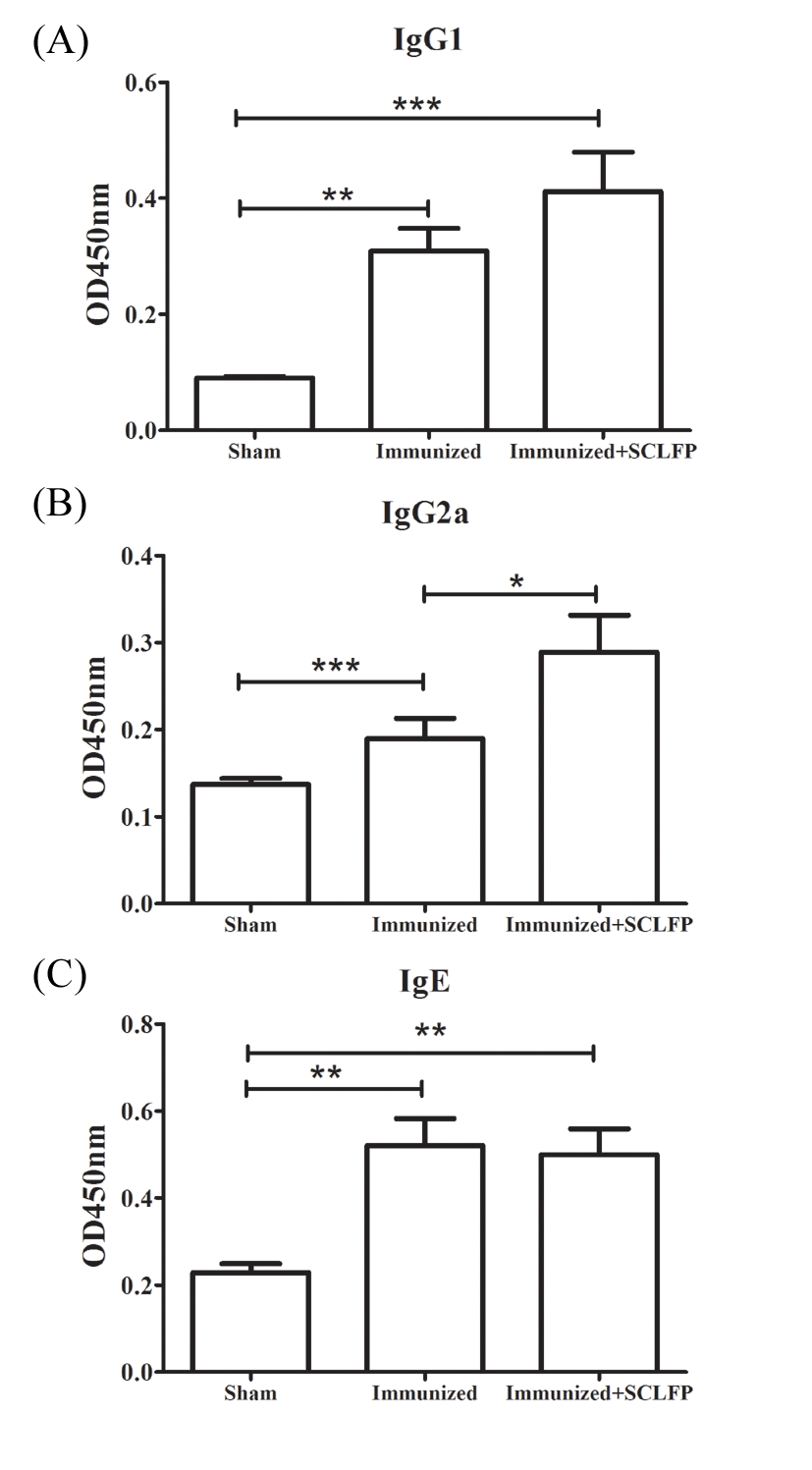

Supplement: Additional file 3: Figure S2 — Serum antigen-specific antibody responses to administration of SCLFP. Sera were collected 8 days after the third epicutaneous sensitization, and OVA-specific (A) IgG1, (B) IgG2a and (C) IgE antibody levels were determined by ELISA. Data are represented the mean ± SEM and performed one-way analysis of variance (ANOVA) followed by Tukey’s multiple comparison test. All p-values less than 0.05 were considered statistically significant. [file 1472-6882-14-194-S3.tiff]
